# Quality improvement collaboratives and the wisdom of crowds: spread explained by perceived success at group level

**DOI:** 10.1186/s13012-014-0091-2

**Published:** 2014-07-22

**Authors:** Michel L A Dückers, Peter P Groenewegen, Cordula Wagner

**Affiliations:** NIVEL—Netherlands Institute for Health Services Research, Utrecht, The Netherlands; Impact—National knowledge and advice centre for psychosocial care concerning critical incidents, Diemen, The Netherlands; Department of Sociology, Department of Human Geography, Utrecht University, Utrecht, The Netherlands; EMGO Institute for Health and Care Research, Free University Medical Centre, Amsterdam, The Netherlands

**Keywords:** Quality improvement collaboratives, Quality of healthcare, Hospitals, Dissemination, Diffusion of innovation, The Netherlands

## Abstract

**Background:**

Many studies have been conducted to evaluate the impact of quality improvement collaboratives (QICs) on the quality of healthcare. This article addresses an underexplored topic, namely the use of QICs as ‘intentional spread strategy.’ Its objective is to predict the dissemination of projects within hospitals participating in a change programme based on several QICs. We tested whether the average project success at QIC level (based on opinions of individual project team leaders) explains the dissemination of projects one year later.

**Findings:**

After one year, 148 project team leaders of 16 hospitals participating in the two-year programme were asked to rate the success of their improvement project on a scale from 1 to 10. At the end of the second year, the programme coordinator of each hospital provided information on the second-year dissemination. Average success scores and dissemination statistics were calculated for each QIC (N = 12). The non-parametric correlation between team leader judgment and dissemination rate at QIC level is 0.73 (P < 0.01).

**Conclusions:**

Previous work, focusing on the team and hospital level, showed which factors contributed to local success stories. It also illustrated how successes play a role in dissemination processes within programme hospitals. The current study suggests that we cannot ignore the extent to which the dissemination potential of individual projects is defined by their QIC. Aggregated team leader judgments at the QIC level might predict the future dissemination in participating organizations. The findings, however, need to be replicated in larger, independent samples.

**Electronic supplementary material:**

The online version of this article (doi:10.1186/s13012-014-0091-2) contains supplementary material, which is available to authorized users.

## Background

In 2002, Øvretveit *et al.* published their ‘lessons from research’ on quality improvement collaboratives (QICs) [1]. They established the need for more research on different types of QICs and their effectiveness. QICs bring ‘…together groups of practitioners from different healthcare organisations to work in a structured way to improve one aspect of the quality of their service. It involves them in a series of meetings to learn about best practice in the area chosen, about quality methods and change ideas, and to share their experiences of making changes in their own local setting.’ (p. 345) [1].

In the last decade, the effectiveness of QICs in stimulating improvement has been evaluated quite extensively. A systematic review concluded that the evidence underlying QICs ‘…is positive but limited and the effects cannot be predicted with great certainty’ and recommends further research into success factors [2]. Recent studies examined determinants for success or failure [3–7]. Yet, researchers focussed less on the function that Øvretveit *et al*. labelled ‘an intentional spread strategy’ [1]. This function is the topic of this article, which is based on data from an independent evaluation of a change programme for hospitals in the Netherlands.

Between 2004 and 2008, 24 hospitals joined a ‘multilevel quality collaborative’, a two-year programme based on a variety of QICs and a leadership programme for executives. In the first year, multidisciplinary teams comprised of health care professionals and managers from each hospital participated in the QICs and implemented improvement projects, pursuing different targets. In the second year, projects were disseminated over new units and patient groups within the hospitals (see Table 1). Whilst implementing the projects, hospitals were expected to enhance their internal quality improvement infrastructure [8,9].

**Table 1:** **Objectives and planned number of projects per hospital**

**Project**	**Objective**	**Number of planned projects per hospital**	
		**Year 1**	**Year 2**
*Patient logistics*			
Waiting lists	Access time for outpatient appointment is less than a week	2	2
Operating theatre	Increasing operating theatre productivity by 30%	1	1
Process redesign	Decreasing the total duration of diagnostics and treatment by 40-90% and length of in-hospital stay by 30%	2	2
*Patient safety*			
Medication safety	Decreasing the number of medication errors by 50%	2	2
Pressure ulcers	Percentage of pressure ulcers is lower than 5%	2	2
Postoperative wound infections	Decreasing postoperative wound infections by 50%	1	0
**Total**		**10**	**9**

(Source: Dückers *et al.*) [9].

Before presenting our research question, we provide a brief overview of relevant earlier findings from the programme evaluation.

## Previous findings

**Figure 1:**
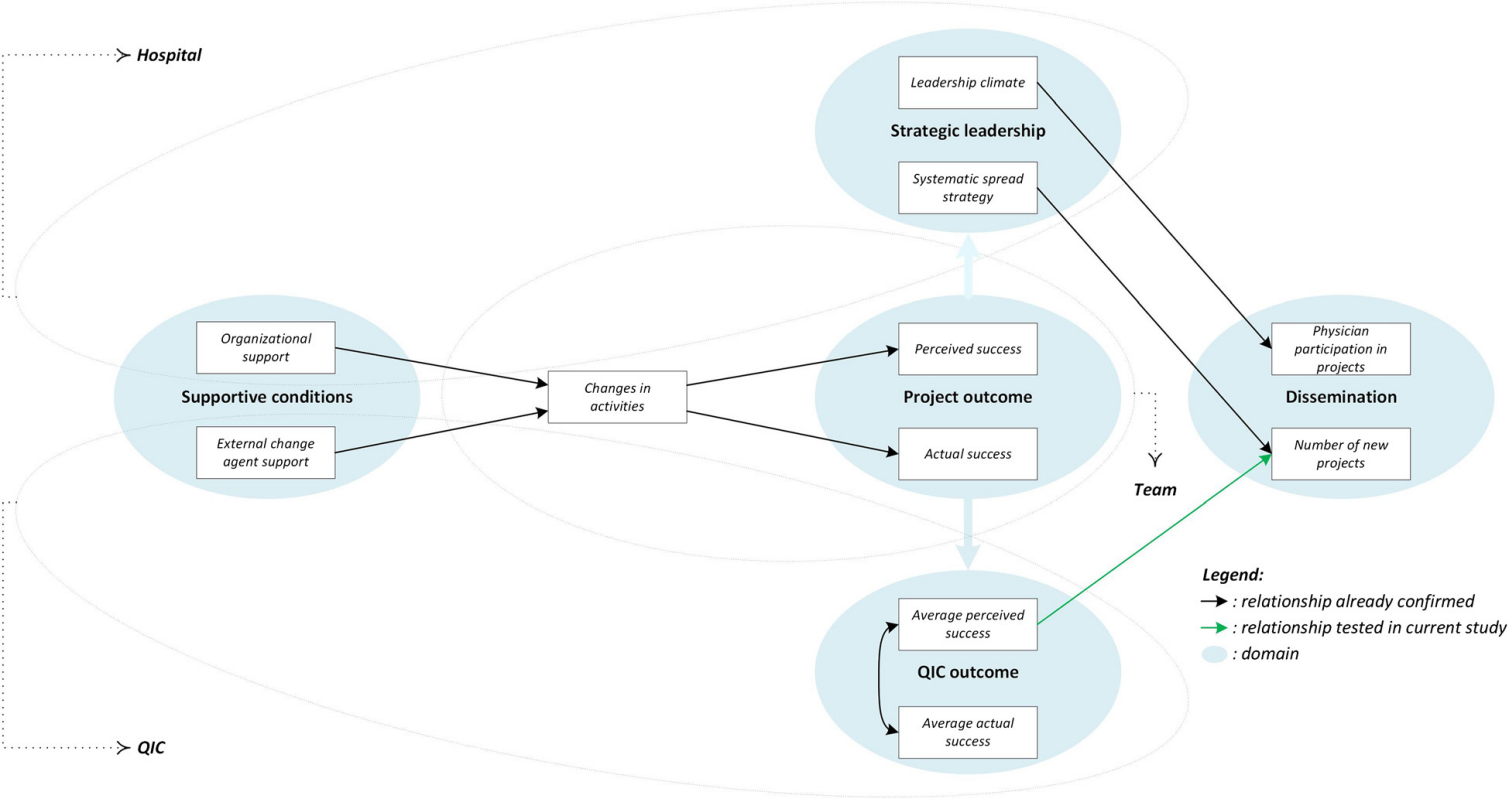
**Overview of relationships.**

The black lines in Figure 1 represent the relationships between several domains that we assessed in other studies.

### Between strategic leadership and dissemination

Hospitals adopted a systematic strategy for spread and sustainability based on learning from successful first-year projects. Projects judged positively were more likely to be disseminated than projects viewed less positively or even negatively [9]. We also confirmed the relevance of the strength of the leadership climate. In hospitals where more physicians agreed upon the extent to which CEOs stimulated improvement, these physicians participated in more improvement projects and vice versa [10].

### Between supportive conditions and first-year project success

As expected, actual and perceived project success depended on the support from the hospital organization and external change agents (trainers and advisors who operated each QIC) [7,11]. The influence of both support sources was mediated by the number of changes applied by the teams [7]. In other words, success rates provided by project team leaders say something about realized changes, support provided, and thus about the project’s fit-within-context.

### Between first-year QIC success and dissemination

The current study is placed at QIC level, where we already found a strong association between perceived success and actual success; two outcomes that differed significantly between QICs but not between hospitals [7]. In this short report, we test the (green) relationship between the average perceived success score at the QIC level and the second year dissemination.

### Research question

Although conditions and activities at the team and hospital level could be linked to perceived success and dissemination, the contribution of the QIC is under-researched. Still, teams of the same QIC have something in common. The nature of their improvement topic is similar. Quality goals, change measures and methodology, complexity, and relevance for everyday practice are comparable to a certain degree, as are the potential obstacles (in one process redesign QIC, for instance, teams were hindered by a lack of internal competition and testable concepts, and by the complexity of aligning interests of involved units) [11]. As such, the teams are all exposed more or less to the same temporary external knowledge-exchange-and-support-vehicle aimed at team empowerment. Based on the shared content and process, teams joining a particular QIC have something mutual, explaining the local fit and thus the chances to generate successful projects.

In order to gain a better understanding of the role the QICs played in the dissemination within programme hospitals, this study seeks to answer the following question: do aggregated success scores at the QIC level explain future dissemination of projects?

## Methods

**Figure 2:**
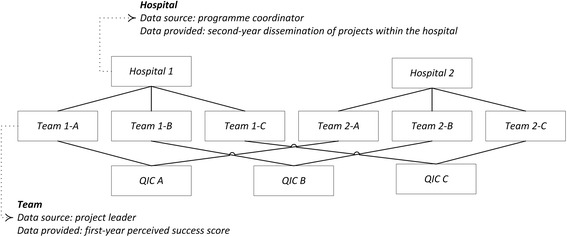
**Teams nested in hospitals and QICs.**

By the end of 2004, a first group of project teams from eight hospitals started with the programme, a second group of eight hospitals started one year later. Study data was collected from two sources: project team leaders (responsible for the implementation of individual projects) and programme coordinators (responsible for the coordinated implementation of the programme in their hospital) (Figure 2). In January 2006 and 2007, 148 project team leaders were asked to rate the overall success level of their project on a scale from 1 to 10. In the last quarter of 2006 and 2007, a questionnaire was sent to the programme coordinator appointed in each hospital for the duration of the programme. The coordinators were requested to fill out a table with the number of units or patient groups where projects were implemented in the second year (the same table as the one described by Dückers *et al*.; see the Additional file 1) [9]. Based on these data, an average perceived project success and an average second-year dissemination rate were calculated for each of the 12 QICs (six project types, two hospital groups). Associations and differences between groups were tested in SPSS 20.

The study protocol was approved by the Medical Ethics Review Committee of the VU University Medical Centre (registered with the US Office of Human Research Protections as IRB00002991). The study does not fall within the scope of the Medical Research Involving Human Subjects Act.

## Results

A total of 84 project team leaders (57%) rated the success of their project and 15 programme coordinators (94%) filled out the dissemination table. Table 2 contains general descriptive information on the data (*i.e.,* mean, median, inter-quartile range, minimum and maximum values) and the average QIC scores. Each average perceived success is accompanied by the number of project team leaders who rated their project’s success level (between parentheses). The highest average perceived success was found among medication safety, process redesign and waiting list projects. The operation theatre QICs reveal the lowest average success and dissemination rare. The dissemination differs significantly between the twelve QICs (Kruskal-Wallis test; p < 0.001) and between both hospital groups (Mann–Whitney U test; p < 0.05). The non-parametric correlation between perceived success and dissemination at QIC level is 0.73 (Spearman’s rho; p < 0.01).

**Table 2:** **First-year perceived project success and second-year dissemination: distributional information and QIC averages**

	**Distributional information**				**First group of hospitals**						**Second group of hospitals**					
	**Mean**	**Median**	**IQR**	**Min-Max**	**WL**	**OT**	**PR**	**MS**	**PU**	**PW**	**WL**	**OT**	**PR**	**MS**	**PU**	**PW**
Perceived project success*	6.58 (84)	7.05	1.69	4-8	7.25 (8)	5.0 (3)	7.55 (11)	8.00 (7)	6.90 (7)	6.1 (5)	7.33 (12)	4.00 (4)	7.63 (8)	7.19 (8)	5.71 (7)	6.25 (4)
Second-year dissemination**	4.63 (15)	4.09	5.85	0.14-11.88	7.13 (8)	0.63 (8)	6.88 (8)	11.88 (8)	6.88 (8)	3.75 (8)	4.43 (7)	0.14 (7)	3.14 (7)	7.57 (7)	2.29 (5)	0.86 (6)

Notes: IQR = Inter-quartile range, Min = Minimum, Max = Maximum, WL = Waiting lists, OT = Operating theatre, PR = Process redesign, MS = Medication safety, PU = Pressure ulcers, PW = Postoperative wound infections.

*Between parentheses, total number of project team leaders providing a success score.

**Between parentheses, total number of programme coordinators providing dissemination data.

## Conclusions

The programme provided a unique opportunity to test whether project successes at the QIC level, based on opinions of individual project team leaders, are indicative for dissemination. The study supports the potential of QICs as multi-organizational intentional spread strategy. QICs are a platform to reach professionals and managers from different organizations. The ability of QICs to generate team success stories appears to have an influence on the intensity of the spread. However, in the end, successful dissemination depends on the capacity of the teams’ supportive home organizations to establish an optimal receptive context [12,13]. Organizational readiness suggests that people at all levels have sufficient motivation, capability, and opportunity to implement or facilitate necessary changes [14]. This is why different authors recommend a multilevel approach for large-scale implementation and spread [6,15]. In case of the multilevel quality collaborative, this proved beneficial. What this study adds to our earlier work is an explanation for the impact of QICs on dissemination processes in organizations. Programme developers, managers, and evaluators are invited to look at what individuals have to say, but also to take the ‘wisdom of crowds’ into account [16]. Group data can be informative. The findings open other research avenues to explore, for example, on how policymakers and QIC planners can gather and use information on perceived success when conducting QICs.

Several limitations must be mentioned. Mittman emphasized how expectation biases and belief perseverance produce systematic overweighting of evidence and observations by respondents in QIC evaluations [17]. This is a typical risk [7]. The small sample, moreover, makes it necessary to be cautious with interpreting the results and should be seen as an encouragement to replicate the study in larger, independent samples. It is possible that actors within the second group of hospitals were exposed to or influenced in their dissemination decision-making by developments in and results from the first eight hospitals. Other issues we could not take into account are differences in the relative complexity of projects; the extent to which teams chose suitable interventions for their situation and if the same interventions were actually applied on or applicable to the new units and patient groups (which is in fact a broader operationalization of spread than the merely quantitative one in this article); the history hospitals had with the projects before joining the programme; and the possibility that not all projects were completely finished after year one. It is equally noteworthy that the dissemination potential is likely to depend on hospital size and variety in suitable patient groups, specialities, and locations. Consequently, the dissemination magnitude will likely differ between hospitals.

## Additional file

Additional file 1
**Dissemination table.**
