# Supplementary material for: Quality improvement collaboratives and the wisdom of crowds: spread explained by perceived success at group level
Source: Implement Sci. 2014 Jul 22;9:91. doi: 10.1186/s13012-014-0091-2 (PMC4423633; doi:10.1186/s13012-014-0091-2)
Supplement: Additional file 1 — Dissemination table. [file 13012_2014_91_MOESM1_ESM.doc]

**DISSEMINATION TABLE**

Please register the number of units or patient groups where projects were implemented in the first and second year. Was the project implemented in all relevant locations in the hospital?

| ***Project*** | **Year 1** | **Year 1** | **Year 2** | **Year 2** |
| --- | --- | --- | --- | --- |
|  | **No. of units or patient groups** | **Hospital wide** | **No. of units or patient groups** | **Hospital wide** |
| pressure ulcers | ............ | Yes/No | ............ | Yes/No |
| medication safety | ............ | Yes/No | ............ | Yes/No |
| operation theatre productivity | ............ | Yes/No | ............ | Yes/No |
| post-operative wound infections | ............ | Yes/No | ............ | Yes/No |
| process redesign | ............ | Yes/No | ............ | Yes/No |
| working without waiting lists | ............ | Yes/No | ............ | Yes/No |
